# Supplementary material for: Alternative Splicing and Alternative Polyadenylation-Regulated Cold Stress Response of Apis cerana
Source: Insects. 2024 Dec 19;15(12):1006. doi: 10.3390/insects15121006 (PMC11677483; doi:10.3390/insects15121006)
Supplement: Supplementary file 1 [file insects-15-01006-s001.zip › insects-3356587-supplementary File SI.pdf]

File S1. Full sequence of LOC107996313_novel06, LOC107996313_novel10, LOC108000663_novel06, LOC107995758, LOC107998211, LOC108003581 in mRNA.

>LOC107996313

AATGATCCTTTGAAAGTTTTACCGTTGGATTTTAAGAACTGAGATTTGAATCGGATTCCACATTTATCGA
TAAATTTCGAGTTTTCATAAACTTGTCAATTACTATTAACTTGAAGCAAAACTTAACAGTGTGATTTAAA
TTGGTTATAAATAGTACATATAATTATAGACAAATTTATAATCGATAAAACATGTCGAAGGCTCCAGCAG
TTGGAATTGATTTAGGTACTACTTATTCTTGCGTGGGTGTTTTCCAGCATGGAAAAGTTGAAATTATTGC
AAATGACCAAGGAAATCGAACGACACCGAGTTATGTCGCCTTCACGGACACAGAAAGATTAATTGGC
GATGCGGCCAAAAATCAGGTCGCAATGAACCCGAACAACACTATTTTTGATGCGAAAAGATTGATCGG
TCGTCGGTTCGATGATACAACAGTACAAAGTGATATGAAACATTGGCCATTCACGGTGATGAACGATG
GAGGAAAACCAAAGATCAAGGTTTCGTATAAAGGTGAAACAAAGACTTTCTTCCCCGAAGAAGTGTC
TTCGATGGTACTCACGAAAATGAAGGAAACTGCAGAAGCGTACTTAGGAAAAATAGTTACCAATGCCG
TTATTACTGTCCCCGCATATTTCAATGATTCACAAAGACAAGCTACCAAAGATGCGGGAGCGATAGCAG
GCTTAAACGTTTTAAGAATAATTAATGAACCAACCGCGGCGGCGATTGCTTATGGCTTAGATAAAAAA
CTGCCGGTGAAAAGAATGTACTGATCTTTGATTTGGGAGGTGGCACCTTTGATGTGTCCATATTGACCA
TCGAAGATGGCATCTTTGAAGTGAAGTCAACAGCGGGCGATACTCATCTTGGTGGTGAAGACTTCGAT
AATCGGATGGTGAATCATTTCGTTCAAGAGTTCAAGAGAAAATACAAGAAAGACTTAAGTTCGAATAA
GAGGGCCCTTAGACGATTGAGAACTGCTTGTGAGAGAGCAAAGAGAACTTTAAGCTCTTCGACACAA
GCCAGTATTGAAATCGATTCTCTTTTCGAGGGCATTGATTTCTACACATCGATTACCAGAGCCAGATTTG
AAGAACTTTGCGCGGATCTATTCAGAAGTACACTGGAACCGGTTGAAAAAGCTTTGAGAGATGCCAA
GATGGACAAAGCTCATGTTCATAGTATCGTTTTAGTCGGTGGTTCTACCAGAATACCAAAGATTCAAAA
ATTACTACAGGACTTTTTCAATGGAAAAGAATTGAACAAATCGATCAATCCTGACGAAGCTGTTGCTTA
TGGCGCAGCTGTACAGGCCGCTATTTTGCATGGTGACAAATCTCAAGAAGTTCAGGATCTGTTGTTACT
GGATGTCACTCCATTGTCTCTGGGTATAGAAACAGCTGGTGGTGTAATGACAACTTTGATCAAGAGGA
ACACGACGATACCAACCAAGCAAACGCAAACCTTTACTACCTATTCCGATAATCAACCTGGCGTCTTG
ATTCAAGTATACGAAGGAGAGAGAGCTATGACAAAAGATAATAATATTCTTGGAAAGTTTGAACTTACT
GGCATTCCACCTGCACCTAGAGGTGTACCTCAAATTGAGGTCACATTTGACATCGATGCTAATGGTATT
CTGAATGTATCCGCAATTGAAAAATCAACCGGAAAGGAGAACAAGATCACCATCACAAATGATAAAGG
TCGATTGTCGAAAGAAGATATCGAGAGAATGGTGAACGAAGCAGAAAGATATCGTAATGAAGATGAA
CAACAACGTGAAAGAATAACTGCCAAAAATGCATTAGAATCTTATTGTTTCAATATGAAGAGTACAATG
GAAGATGAGAAGATCAAAGATAAGATCGATTCAACGGAAAAAGAAAAGGTTATCAATAAGTGCAACG
AAGTGATTTCATGGTTGGATGCAAATCAATTGGCAGAAAAAGAAGAATTCGCTGATAAGCAGAAAGA
ACTAGAATCCGTTTGCAATCCTGTTGTCACTAAATTATATCAGGGAGGAGCAACACCTGGAGGGTTCCA
TCCAGGTGCTGCTACAGGAGGCGGTGGTGCTGGAGGACCAACCATCGAAGAAGTAGACTAAAAAAA
CAAGAAATCATCCATAACATTAAATATCTTTTTCGTTTCATTGTATTTATTTCAACTCAAACTTTGCACAG
TCATGATCATCGATTTTCATATTTGTTTACATTGATAATGCATATTTTTTGAGATCAGAGACTTTATTTCCT
GCAAGAAAGAGATTTTATTTCTTGTATTTGAAAAGATTGGTAATATAAATTTGTTATAAAAAAAAAAAA

>LOC107996313_NOVEL06

AGACAGAGTTTGGAAAGCGAGAAGTGAACATTGCGTTTACGTATAATTTATTATATTGCGATTTATTCTG
TGATATATACAAAACGAACGATAGAAAAGCGATAAAACATGTCGAAGGCTCCAGCAGTTGGAATTGAT
TTAGGTACTACTTATTCTTGCGTGGGTGTTTTCCAGCATGGAAAAGTTGAAATTATTGCAAATGACCAA
GGAAATCGAACGACACCGAGTTATGTCGCCTTCACGGACACAGAAAGATTAATTGGCGATGCGGCCA
AAAATCAGGTCGCAATGAACCCGAACAACACTATTTTTGATGCGAAAAGATTGATCGGTCGTCGGTTC
GATGATACAACAGTACAAAGTGATATGAAACATTGGCCATTCACGGTGATGAACGATGGAGGAAAACC

AAAGATCAAGGTTTCGTATAAAGGTGAAACAAAGACTTTCTTCCCCGAAGAAGTGTCTTCGATGGTAC
TCACGAAAATGAAGGAAACTGCAGAAGCGTACTTAGGAAAAATAGTTACCAATGCCGTTATTACTGTC
CCCGCATATTTCAATGATTCACAAAGACAAGCTACCAAAGATGCGGGAGCGATAGCAGGCTTAAACGT
TTTAAGAATAATTAATGAACCAACCGCGGCGGCGATTGCTTATGGCTTAGATAAAAAAACTGCCGGTGA
AAAGAATGTACTGATCTTTGATTTGGGAGGTGGCACCTTTGATGTGTCCATATTGACCATCGAAGATGG
CATCTTTGAAGTGAAGTCAACAGCGGGCGATACTCATCTTGGTGGTGAAGACTTCGATAATCGGATGG
TGAATCATTTCGTTCAAGAGTTCAAGAGAAAATACAAGAAAGACTTAAGTTCGAATAAGAGGGCCCTT
AGACGATTGAGAACTGCTTGTGAGAGAGCAAAGAGAACTTTAAGCTCTTCGACACAAGCCAGTATTG
AAATCGATTCTCTTTTCGAGGGCATTGATTTCTACACATCGATTACCAGAGCCAGATTTGAAGAACTTT
GCGCGGATCTATTCAGAAGTACACTGGAACCGGTTGAAAAAGCTTTGAGAGATGCCAAGATGGACAA
AGCTCATGTTCATAGTATCGTTTTAGTCGGTGGTTCTACCAGAATACCAAAGATTCAAAAATTACTACA
GGACTTTTTCAATGGAAAAGAATTGAACAAATCGATCAATCCTGACGAAGCTGTTGCTTATGGCGCAG
CTGTACAGGCCGCTATTTTGCATGGTGACAAATCTCAAGAAGTTCAGGATCTGTTGTTACTGGATGTCA
CTCCATTGTCTCTGGGTATAGAAACAGCTGGTGGTGTAATGACAACTTTGATCAAGAGGAACACGACG
ATACCAACCAAGCAAACGCAAACCTTTACTACCTATTCCGATAATCAACCTGGCGTCTTGATTCAAGTA
TACGAAGGAGAGAGAGCTATGACAAAAGATAATAATATTCTTGGAAAGTTTGAACTTACTGGCATTCC
ACCTGCACCTAGAGGTGTACCTCAAATTGAGGTCACATTTGACATCGATGCTAATGGTATGTGCATTATC
TCTTTTCAATACAACATTTCTTTATAAAAATATCTTCAATATATTCATTAATAAATTAATAAACTTTCTTTT
CACACACATTATAGGTATTCTGAATGTATCCGCAATTGAAAAATCAACCGGAAAGGAGAACAAGATCA
CCATCACAAATGATAAAGGTCGATTGTCGAAAGAAGATATCGAGAGAATGGTGAACGAAGCAGAAAG
ATATCGTAATGAAGATGAACAACAACGTGAAAGAATAACTGCCAAAAATGCATTAGAATCTTATTGTTT
CAATATGAAGAGTACAATGGAAGATGAGAAGATCAAAGATAAGATCGATTCAACGGAAAAAGAAAAG
GTTATCAATAAGTGCAACGAAGTGATTTCATGGTTGGATGCAAATCAATTGGCAGAAAAAGAAGAATT
CGCTGATAAGCAGAAAGAACTAGAATCCGTTTGCAATCCTGTTGTCACTAAATTATATCAGGGAGGAG
CAACACCTGGAGGGTTCCATCCAGGTGCTGCTACAGGAGGCGGTGGTGCTGGAGGACCAACCATCGA
AGAAGTAGACTAAAAAAACAAGAAATCATCCATAACATTAAATATCTTTTTCGTTTCATTGTATTTATTT
CAACTCAAACTTTGCACAGTCATGATCATCGATTTTCATATTTGTTTACATTGATAATGCATATTTTTTGA
GATCAGAGACTTTATTTCCTGCAAGAAAGAGATTTTATTTCTTGTATTTGAAAAGATTGGTAATATAAAT
TTGTTAT
>LOC107996313_NOVEL10
GTACAAAGTGATATGAAACATTGGCCATTCACGGTGATGAACGATGGAGGAAAACCAAAGATCAAGG
TTTCGTATAAAGGTGAAACAAAGACTTTCTTCCCCGAAGAAGTGTCTTCGATGGTACTCACGAAAATG
AAGGAAACTGCAGAAGCGTACTTAGGAAAAATAGTTACCAATGCCGTTATTACTGTCCCCGCATATTTC
AATGATTCACAAAGACAAGCTACCAAAGATGCGGGAGCGATAGCAGGCTTAAACGTTTTAAGAATAAT
TAATGAACCAACCGCGGCGGCGATTGCTTATGGCTTAGATAAAAAAACTGCCGGTGAAAAGAATGTAC
TGATCTTTGATTTGGGAGGTGGCACCTTTGATGTGTCCATATTGACCATCGAAGATGGCATCTTTGAAG
TGAAGTCAACAGCGGGCGATACTCATCTTGGTGGTGAAGACTTCGATAATCGGATGGTGAATCATTTCG
TTCAAGAGTTCAAGAGAAAATACAAGAAAGACTTAAGTTCGAATAAGAGGGCCCTTAGACGATTGAG
AACTGCTTGTGAGAGAGCAAAGAGAACTTTAAGCTCTTCGACACAAGCCAGTATTGAAATCGATTCTC
TTTTCGAGGGCATTGATTTCTACACATCGATTACCAGAGCCAGATTTGAAGAACTTTGCGCGGATCTAT
TCAGAAGTACACTGGAACCGGTTGAAAAAGCTTTGAGAGATGCCAAGATGGACAAAGCTCATGTTCA
TAGTATCGTTTTAGTCGGTGGTTCTACCAGAATACCAAAGATTCAAAAATTACTACAGGACTTTTTCAAT
GGAAAAGAATTGAACAAATCGATCAATCCTGACGAAGCTGTTGCTTATGGCGCAGCTGTACAGGCCGC
TATTTTGCATGGTGACAAATCTCAAGAAGTTCAGGATCTGTTGTTACTGGATGTCACTCCATTGTCTCTG

GGTATAGAAACAGCTGGTGGTGTAATGACAACTTTGATCAAGAGGAACACGACGATACCAACCAAGC
AAACGCAAACCTTTACTACCTATTCCGATAATCAACCTGGCGTCTTGATTCAAGTATACGAAGGAGAGA
GAGCTATGACAAAAGATAATAATATTCTTGGAAAGTTTGAACTTACTGGCATTCCACCTGCACCTAGAG
GTGTACCTCAAATTGAGGTCACATTTGACATCGATGCTAATGGTATTCTGAATGTATCCGCAATTGAAAA
ATCAACCGGAAAGGAGAACAAGATCACCATCACAAATGATAAAGGTCGATTGTCGAAAGAAGATATC
GAGAGAATGGTGAACGAAGCAGAAAGATATCGTAATGAAGATGAACAACAACGTGAAAGAATAACTG
CCAAAAATGCATTAGAATCTTATTGTTTCAATATGAAGAGTACAATGGAAGATGAGAAGATCAAAGATA
AGATCGATTCAACGGAAAAAGAAAAGGTTATCAATAAGTGCAACGAAGTGATTTCATGGTTGGATGCA
AATCAATTGGCAGAAAAAGAAGAATTCGCTGATAAGCAGAAAGAACTAGAATCCGTTTGCAATCCTGT
TGTCACTAAATTATATCAGGGAGGAGCAACACCTGGAGGGTTCCATCCAGGTGCTGCTACAGGAGGCG
GTGGTGCTGGAGGACCAACCATCGAAGAAGTAGACTAAAAAAACAAGAAATCATCCATAACATTAAAT
ATCTTTTTCGTTTCATTGTATTTATTTCAACTCAAACTTTGCACAGTCATGATCATCGATTTTCATATTTGT
TTACATTGATAATGCATATTTTTTGAGATCAGAGACTTTATTTCCTGCAAGAAAGAGATTTTATTTCTTGT
ATTTGAAAAGATTGGTAATATAAATTTGTTATAAAAAAAAAAAAATCACTGTGTTTAAATATTTATTTCTAT
ATTGTGTCTATTTATTAATTTTTTTAATTGATTTATCAATGTTTTTTGTTTTTGATATTTATAAATTTCTAATT
CTTGTGATTCTTTATTTTTGAAAAATGCTATCTTGTTAAATTAAGTTGAACGTTGGTATTCAGTACAAATT
CGCTGTAATCCAATTTTATTTATCTCTCGATGGATCAGCGTATATATCCAACATCAAAGTGAACTATTATA
AATTATCCAAATCACGTTATTCAGGAATATGAAACAGCTTCGAGACCACCATTATAAAAAGAATTTGTA
AAACATAACAATAGATCTATGCCATAAATGTCGTTTACTATCATTATTACATAATATAGAACTTAATTCTAC
AAGAATCGCAATTTAATCGAGAGATAAGTGCATAAATGGTGCATCATTACGCATCATTTTTAATAATAGC
ATTTTAATAATAGAATGAGGGACGGAAACAAATTTCTTTTTTTAAAATAAAAGACGAAATGTTCAAAAT
GACGTACGCTCTATCCAATTTACGTCACTCACTCTAAATACAAGAATTAAGGCTAATATCTCGTGCCATT
GTGCAGTCTCCTAATTTTTTTGATTTTCAATACAATCCAACAGATCGTTACTTTTTCATCCAACTGTTTG
ATCATCTCTATTATCTTGATTACTCTAAATTTGTGGCATATCATGTATAACGGCATCGAACACGAAACTAT
TCATTAAAGTTTGCGTATAATAAAGAAGTAGATCGGATGCAAAGATTTGATCAGGTGAAATAATAATAA
AGAACGTGAAGCATTGATATTGGACCGCATAATATAACTATAAAAATGGCCAAGATTTGACTCATCTTC
ACGTATCCATCTTCGATCAATTCTTTGCCGGTATTCTTATCCAATATCTTCATCAATTTAGGCCATCTGTC
ATAATGTCTACATGGTAAACGTAATAATATGCTGCAAGATGACACACGGTTTCGTTCATGAAATACATGA
TTTTGAACAATTCATTGGTGAAATTGGCCTTGCTGAAATTGGCGAAATCCATTGCGCTAAATAGGACGA
TCGAGCAAACGCTGAAGTGAATCATTAGAGAGATGACAGTGATCCATTTTGAACAATCGTGCAACTCC
AACACCTAACAACCAAGAAACATACATTATCGGACGTAATATACATTTTGGAATCGATGGATCCCCTATT
CGAACGTTTATCTCTTCCTCCATGATGATTTATTCTTCCAGAAACGAGCAGAAGTCTCGATTCTCGATTT
GAAAATTTCCTCGAGGTGAGATAGCTTTAACTCGAAAGCAAAGCAAAACACGCTGAATTCTTGATCCG
AGGATTTGCATCTCACATATTCGTCTTTTAATAGACAAAGATTGAACGCGATACGAGATTCGTTTCGCGT
TCATATCGGAATTCAAGAAAAGTTGGGGAAGATTTATGCATGGCAACACCATCAAGGCACTTTCATTTT
TTCGATGGAAGTAGAATAGGCATGAAATTAAACGCACGAAAGGACACGAATGGGACATCATTTGTTCC
GTGATATGTGTGTATATATCGAAAAAACGAGCTTTATTTGCCCAACGACTATATTACAATTTTATTATACA
GTAAACATTTAAAATGGAATAAAACGATAACTTTG

>LOC108000663

TTTATCAATAATTATTTAAAAACAGCATACGATCATTTTTGTTCTTCATTCTGAATGTCATGTATCATTTAA
ATGCGCGAAAAACAAACGAGTTCTCGAAAGAGTCCAACAGAGAGGAACGTTCTAGTAGCCTCTAGTC
TATTCATTCGGTTGCTCTCGTGACGATTCGGCCATACTCGACATAGCAGTCACGTGGCTTTCTACAACC
CACACAATAAATATATTTAAGTTGCTCCGGGAACGCACTGACAGTTTCACTATTGACCCGAAGGGAATA
TGTTTTATAACGCATATATATTGACGATTTTGTTGAAAAGACGTTTTTTTTTTTCAATAAAAAATTTTTAT

ATGCCAGTGTGTGATACAAAGTGTAAAAAATAGACGACTAATGTCAATAGTGATCATATTATATAACGA
AAGAAAGAAAATAAAAAGCTAAAATACTAGCGAGGAGATTGAAAGGAAGAATAAGAATAACTGTCAC
ACGTTTTCTTGGTAATATTACAAGATTTTTAAAAAAAGAAAACGAAAGTGTGTTTTAAATCATTTATTTT
TCGTTCTGCCTTTTTTTGTTAACACATCGGCATTGTTTAGTGAGCTTTTTGGCTGATTCTTTGAAAACGT
AAAAAAAAAAAACATTTAACCTGCAGAGCAAGTTCTTAACGGCAAAAGGGCACGAACTTAACCTTCT
AGTGTGTGAAAAAAAGCCAACATATATCTATATTAAAAAGTTTCATCCTTTGAGAGAAAAAAGGAAAA
GGACACCACGTATTCGAAATCAGGCACGGATCAAAGGGTCCACAAAATGGACTCGCCTGTCATCGTGG
ACAAAGCATCTGAATTCGGAGAATCAATTGATTTGGATCGTCCATTTCCCGGATTTCCATTCGACGGCG
ACAATTTTGGTCGACGCAGTGATATTCGTGCCCATCTGGATGATTTAGCAGCTCGTCATCCAGAATTTG
CAGATCATTTATTAGGTCCACCTTGGGGTGATATCCCTTTCCACAATTCTTTTCGCAATCGAAACCGTGA
TATTGGAAATAAAGAACGTAATAATTATCAACAAGGCTATTCCGACGAGGACGCAAGAAGTCAGGCAA
GCGGAAGTAGTGTTGCCAGTGGAACTAGTGTCTCCAGTTCTCACGGCGAATCAGACGTTAATCAGAAT
CAACAAAACAAATCTCCAAATTTTGAGCAAAATATTAAAAAAAGTCAAATTCCACAATACGGATTACG
CAACACAGTAGATATAGGTCAACATCATCATAACATGGAGAATACTGATAAAGGAAATCGTGGTCAACG
TTCTATGTCTGCACCTCCAGAAAATAGACAATCTTTTAATCAACAATTTCAGCAACAAGAACAACAATC
ACCACAGCAATCACAGAGATATGTTTCCAGGATAGATATCACACCACAGCATAATCAGCCTCAACATAA
ATCACAACAACAATCTCAACAGCAACAACAACAAAAACCGCAACAACAAAGCAATGTTAGGCATATT
CCAATTTTTGTTGAGGGCAGAGATGAACCTGTGCTTCCAAGGAGTTTCGATCAAGGTTCGCAACATCG
AACTTTTGTGAATATGGATCCACATTTTCAAAGACCGACGCCATTTGGTCAGCATTTTGCTGGAAGACA
TCAACAATGGTCTCCACATTTTCAAGAATCGTTTTATCAGCAGCCAAGTGCATTTGAACATCCCTCTGT
GAGACAACAGCAAACTCATAATTTTAAAGAGCCTAGACAACAATTGTACAATGAAAGACAACCTCAG
CATCAGCAATATTGTGAAAGACAACCTCAGCAACAGCATTATGAGCAACAAAAACAACAAAAAACTC
AACAGCCTCAACAACAGAGACAAGAGTTACCTATTGTTTTAAAGGATCCTTTAGAGAGAGTAGCTCTT
GTTCAAAAAGAAGTTAATGCTTTGGCTGAACAAGTCAAGCAATATAATGGCATTTCAAGAACAGATAA
AGAATATATTTATTTAGATGAAATGCTTACTAGAGAATTGATTAAATTGGACGATATTGAAACAGAAGGT
AGAGACAATGTGCGACAGGCACGTAAAAACGCAATAAAAAGTATACAGGAAACGATTAGCTTACTGG
AGTCGAAAGCGCCACTGCCTTTGCAACAAATGTCAGCAGAAGAACAGGAAAAACAGGAAGATCAAG
TTTCCAACATTACTGAAGAGACTGAAAAAATGAATCAGAAACTAGAAGATCAATCACAGAATAAGGA
AGCTATCCCACTGCCTCCTGGTCCATCATCTCCAATGAAAAAGACTGGAGAAATCATCGAAACTTGTGT
GGAAAAATTAGAAAGTTTTGATAATAATCAAACGGCAGATTTACAAGAGGAAGCTAATCAAACTTCCA
ACAAATCTATAAATGCGATTTCAAATGCATTGGAAAAACAGGAAATGATTGCAATGGATACACATAGTT
CAGAAGAATCAAATATAACTGAAAAGAAGAAATTGAAAAATGTTTCTGAAGAGAAAAAACAAAAATA
TATCCCTGAAGAGAAAAAAGTAGATATTTCTATTGAGAAACCTATAAAGAATATTTCTTCCGAGAATAA
GTCAGAAAATCTTTTAGATGAAAAAAAAATAGAGAACATTTCTGATCACAAGATAGAAAACACTACTA
TGGAAATAAAAGAAACTATTGAAAAAAATAAAATTGAAAAAGATGTCAAAGAAGAAAAAGAAACAG
AGAAAACTGATAATAAACAGTCTATTCAAGAAGAACAACAGAAAACAGAAGAGAAAATGGACCTAGA
TAGTGGTGAAATAAAACAATCACCTAAATCGCAGAAGAAAGGAAAAAAGACGAAAAAACAGGTTCC
AATTTCTGATAAACCTATACCATTGCCAGCTCCAGAAAGTACTGAGGCAAAGTAAAATAACAGAAAAA
ATTAGTTACGATTTTAATTGTATTAAAAGATACGTCAATCTAATTATTGTTAGTTTGTGTATCTTTAGAAC
GAAACTATTGTTTTCATTCAAGAAATCACAAAATCTCGTGATCGTTATTTCTTTTCTGTTTTATGATTATT
TTTGGTGCCAGATTGACAAAAACGTCGTGGATCGACGTGGTAATCGCACAATCGTTTATTAGCATAGAA
TCATGCTGATCAAAAACTTCTTCTGTTTTGCTTCGTTATTTTAATTTCCATGTTATTATAATTCAAACAGG
GGAAGCATATATGGAATCTGTTCAATGAATTTAGAGCGTATATAAAAAAAACAACGAATGTAGCATTTT
TAAAGTTGTTTTCGCTGTTGCGTTGCATTTGAACGAGGCTGTCCCCTTTCTTTTCTATATCGCGATTACA

TATGATTTCAAACGAAATCGAATATACAACATTCACCAGATTGTATTATACATTTTGAATGTTTAAATTTT
GCACTTAAAAAAATAAATATTGGTCCTCATTTAAAAATAA
>LOC108000663_NOVEL06
ACTGACAGTTTCACTATTGACCCGAAGGGAATATGTTTTATAACGCATATATATTGACGATTTTGTTGAA
AAGACGTTTTTTTTTTTCAATAAAAAATTTTATATGCCAGTGTGTGATACAAAGTGTAAAAAATAGAC
GACTAATGTCAATAGTGATCATATTATATAACGAAAGAAAGAAAATAAAAAGCTAAAATACTAGCGAGG
AGATTGAAAGGAAGAATAAGAATAACTGTCACACGTTTTCTTGGTAATATTACAAGATTTTTAAAAAAA
GAAAACGAAAGTGTGTTTTAAATCATTTATTTTTCGTTCTGCCTTTTTTTGTTAACACATCGGCATTGTT
TAGTGAGCTTTTTGGCTGATTCTTTGAAAACGTAAAAAAAAAAAACATTTAACCTGCAGAGCAAGTTC
TTAACGGCAAAAGGGCACGAACTTAACCTTCTAGTGTGTGAAAAAAAGCCAACATATATCTATATTAAA
AAGTTTCATCCTTTGAGAGAAAAAAGGAAAAGGACACCACGTATTCGAAATCAGGCACGGATCAAAG
GGTCCACAAAATGGACTCGCCTGTCATCGTGGACAAAGCATCTGAATTCGGAGAATCAATTGATTTGG
ATCGTCCATTTCCCTGATATAAAGGCTGTAATACACATCGTCACGAATGGAATATCCACGCGAAATCATT
TCTTGAAATGGCATGAATTATCGTAATATGAATGTACGAATTGTGACATTGATTACGAATGCTCTTCTAA
AATCGAGAAATATGATCGCAATGGAAATCTTCATATTTTTAAAAACGATTTCACGGATTTCCATTCGACG
GCGACAATTTTGGTCGACGCAGTGATATTCGTGCCCATCTGGATGATTTAGCAGCTCGTCATCCAGAAT
TTGCAGATCATTTATTAGGTCCACCTTGGGGTGATATCCCTTTCCACAATTCTTTTCGCAATCGAAACCG
TGATATTGGAAATAAAGAACGTAATAATTATCAACAAGGCTATTCCGACGAGGACGCAAGAAGTCAGG
CAAGCGGAAGTAGTGTTGCCAGTGGAACTAGTGTCTCCAGTTCTCACGGCGAATCAGACGTTAATCAG
AATCAACAAAACAAATCTCCAAATTTTGAGCAAAATATTAAAAAAAGTCAAATTCCACAATACGGATT
ACGCAACACAGTAGATATAGGTCAACATCATCATAACATGGAGAATACTGATAAAGGAAATCGTGGTCA
ACGTTCTATGTCTGCACCTCCAGAAAATAGACAATCTTTTAATCAACAATTTCAGCAACAAGAACAAC
AATCACCACAGCAATCACAGAGATATGTTTCCAGGATAGATATCACACCACAGCATAATCAGCCTCAAC
ATAAATCACAACAACAATCTCAACAGCAACAACAACAAAAACCGCAACAACAAAGCAATGTTAGGCA
TATTCCAATTTTTGTTGAGGGCAGAGATGAACCTGTGCTTCCAAGGAGTTTCGATCAAGGTTCGCAAC
ATCGAACTTTTGTGAATATGGATCCACATTTTCAAAGACCGACGCCATTTGGTCAGCATTTTGCTGGAA
GACATCAACAATGGTCTCCACATTTTCAAGAATCGTTTTATCAGCAGCCAAGTGCATTTGAACATCCCT
CTGTGAGACAACAGCAAACTCATAATTTTAAAGAGCCTAGACAACAATTGTACAATGAAAGACAACCT
CAGCATCAGCAATATTGTGAAAGACAACCTCAGCAACAGCATTATGAGCAACAAAAACAACAAAAAA
CTCAACAGCCTCAACAACAGAGACAAGAGTTACCTATTGTTTTAAAGGATCCTTTAGAGAGAGTAGCT
CTTGTTCAAAAAGAAGTTAATGCTTTGGCTGAACAAGTCAAGCAATATAATGGCATTTCAAGAACAGA
TAAAGAATATATTTATTTAGATGAAATGCTTACTAGAGAATTGATTAAATTGGACGATATTGAAACAGAA
GGTAGAGACAATGTGCGACAGGCACGTAAAAACGCAATAAAAAGTATACAGGAAACGATTAGCTTAC
TGGAGTCGAAAGCGCCACTGCCTTTGCAACAAATGTCAGCAGAAGAACAGGAAAAACAGGAAGATC
AAGTTTCCAACATTACTGAAGAGACTGAAAAAATGAATCAGAAACTAGAAGATCAATCACAGAATAA
GGAAGCTATCCCACTGCCTCCTGGTCCATCATCTCCAATGAAAAAGACTGGAGAAATCATCGAAACTT
GTGTGGAAAAATTAGAAAGTTTTGATAATAATCAAACGGCAGATTTACAAGAGGAAGCTAATCAAACT
TCCAACAAATCTATAAATGCGATTTCAAATGCATTGGAAAAACAGGAAATGATTGCAATGGATACACAT
AGTTCAGAAGAATCAAATATAACTGAAAAGAAGAAATTGAAAAATGTTTCTG
>LOC107995758
CTTGAAAATGAACTCCATGATATATACCCTCTACGGGTTTGCGATATTTTTCATGATATTCTGGTCAGTGA
TGTGGATTGTTCATGTACTGGCACTGATTGCTGGTCACTGGAAACTACATCGTAAAATCACTCAAGTAC
CAACCTACGAAACGCCCTTACCCGGTGTATCAATCATAAAACCACTAATGGGCGTTGATCCGAATCTAT
TCAGTAATTTAGAGACATTTTTCACCATGCAATACCCACGTTACGAGTTATTATTTTGTGTGGAAGATGA

CTCGGATCCTGTGTTAATGTTAGTGCGTAAATTGATTGAGAAGTATCCAGAAGTGGACGCAAAACTTTT
TATCGGTGGCTGTAATGTAGGTGTAAATCCAAAGATCAACAATATGCAACCAGCATACGAGGCGGCCA
AGCATGAGTTTGTGTTGATCAGTGACAGTGGTATCAAGATGAAAGAGGATACGCTCTTGGATATGATTA
ATTACATGACTGACAATGTTGCATTGGTACATCAAATGCCGTTTACCTGTGATCGTGAAGGTTTTGCCG
CAGCATACGAAAAAATCTTTTTGGTACCGTACAATCGCGTATGTATCTTGCCGCCGATTTACTTAGGAT
AAACTGTCATACCGGTATGTCAGCTTTATTGAGGAAAGCGCCAATTGACGAGGTCGGTGGATTAAAGA
CGTTCGGTGTGTATTTAGCAGAGGACTTTTTCTATGCAAAGTCATTGACCGATCGTGGTTGGCGTATCA
CAGTGTCATCGCAGCCAGCGTTACAAAATAGCGGTCATTGTGAGCTACATTCATTTCAGGCGAGATTAC
GAAGGTGGGCAAAACTGAGGGTAGCTATGTTGCCAACCATGATTGTTCTGGAGCCACTTAGCGAGTGT
TTAGTATTAGGTGCCTGTGCTTCCTGGGCAGCCAGCGTACTTTTTGAGTGGGACTCCCTTGTTTTCTATT
TAGTACACATACTTTTGTGGTTCATGTTCGATTGGACGCTATTGAGCGTCGTGCAAAATGGCCCATTACC
GTTCAATAAGTTAGAATTTGTATGCGGATGGTTACTGAGCGAAATCACCAGGCCTTATCTTTTCCTTCAG
GCAGTTCTAGATCCTCTGATACAGTGGCGATCGCGCGTTTATAAGCTCAGGTGGGGTGGAGTCGCAGA
AGAGGTTAAGGCAAAAGTGAAATATTGAAATATCGTGAAAAATACGTTCTTAATTTTTTTCTTTTCTTTA
TTTCTTTTCTCTTTTTCATCATTCTTTCCTTGTTTTTTATATGTATATACATTCATATACAGATCAACATCCA
CATCAAACTATACGCGTGGGTGTTTTGTAATATCTGATATATTATGCTATTACGCATATAGTACTTATTACG
TTAGGATGAGAAAAAAATATATGTCATATACACAAGAAATGTATTAAACAGAAATATCCGTGCGCGCGT
GTACACATATACGTACATATATATGATATGTTTACATAAAATAGTAATCACGACATGAGTGTATGCACGTG
CAAAGTCATTTTTCAATTATTCTGCATATCATCTCTGCTGGTTGATTCTTAACGGTATGCTTATTTTTTGT
GCAATGTTCTTTGGAAAAAATATAGCATAAAAAAATAAAACATTTTTATTTATGATAGGTTAGAAAAAG
AAAAGATAAAGTAAATAATAATTAATGTACATATATATAATCTTATTTTTTTAAGATCATTGTCCGAAACG
GATAATCTTTATTGCAGTCGTTATTGCAGTATATTCAGCGCAAAATTATTGATACATTCTAATGAAATGAT
TTCTAAAAAAAAAAAAAAATATGATTGCGCAGGTTTCGCGATCAATTTAATATTTTATTGGTATTGTCAT
TATCAGAATATTTTCACGTTCGTTATCTTTATTATTGAAGTATTAATGTTAATATTGTTTTTGATTTGTAAAT
AAATGTTTATTTTTTTCAATATTTGGCATTGAAAATTATATAATAAAGATAA

>LOC107998211
AAAAAAAATTCTACGTAAGCAAAGATTACAATTAGATTGGTACATCCTCACGTGTATAACTTACATAAC
ATAGGTACTGCAAATGTCTTTATCTTAATTCATTTTTAAAAAATGGCTGGCATCACACCTCAAGCTTGCA
GAAATGTATTTCGGCAAGTTAGAGGATTTCATATATCTGCAAATCAAAATGCAGCTGAAGCTAAATTAA
AAAGTTTTGCTGTATATCGTTGGAATCCAGACAAACCTGATGAAAAACCATATATGCAAGAATATAAAG
TTGATCTAAATACTTGTGGTCCTATGGTTTTGGATGCATTGATTAAAATTAAGAATGAAATTGATCCAAC
TTTAACCTTTCGTCGTTCTTGTCGTGAAGGCATTTGTGGTTCCTGTGCTATGAATATTGGTGGTACAAAC
ACATTAGCATGTATAAGCAAAATTGATACTAATTTAAATTCAACTAGTAAAATTTATCCATTACCACATAT
GTATATAGTAAAAGATTTAGTGCCCGATTTAAATAACTTTTATAATCAGTATAAAAGTATACAACCATGGT
TGCAACGTGGTGATGCAAAGGAGACTGGTGCCAAGCAATATTTACAAAGCGTTGAAGATCGTAAAAA
ATTGGATGGTCTCTATGAATGCATCTTATGTGCTTGCTGCAGTACTTCTTGTCCATCATATTGGTGGAATG
GTGATAAATATTTAGGACCTGCTGTACTTATGCAGGCTTACAGATGGATTATTGATTCGCGTGATAGTAA
AGCAAAGGAACGTCTTGCAAAATTACGAGATCCATATTCAGTATATCGTTGTCATACAATTATGAATTGT
ACTCGTACTTGTCCAAAAGGTTTGAACCCTGGAAAGGCAATTGCAGAAATAAAAAAATTATTATCTAAT
ATCAGTCAGAAACAAAAACCTGATCTTGAAACTGCTATATAAATAAAAGAAAAATATGAAAGTAGTCA
TATATATTTTTCGTTTATTATGATTACATAGAAACTTATATTTGATTAAATAGAAACAAATAAATAGCTATC
CAAATTATTTAATAAAATAATGGAATCTTTTATAAATATCGAATATGTTTTACTATAAATAAAATTCAAATT
CCTTTTAATATA

>LOC108003581

TTTGGTTTCTGATCATTCGATGCAGTTCATCTCGGGAAAGATCGTGTGTAACATTCGGCAGGAGATGTT
TCAATAAAATTTCGATCGAACACATTTGTGACGATATGGCAACGAGATCGCAATGGTTGTTGTTGGGAT
TCGTCGGGACTTGGTTGGGATTCTGCACGGCATGGATGGACGAAAGGCCTGTGCTAGAGTCTCGAGAC
GGAAATCTGTTCATTTCCGCGGCAAAGGATAGGAACATCACCCTGAAGACTCTAGGAAATGGTTATGT
GAATGTGAACGAAATTAATTTGCTTCACGTTGCTACCGCTGCGCAAAGCGCGACGCGTTTGATCGAGA
GATGGAAAACCGGATATCTGGCCGAGGTGGAATCGAATTTACAACGTTTGACACAAATTGTAGAGGGT
CCGGACGGTTTAGAGAAAAGAATAGCTACGATGAGAGGATTTGAAGGGAACACTACGCAACAATCAG
AAATTTTTTCTATAAATCAAACTGCATCAGTGGTGAATATGAAGATACGGATGCTACATCATCGAATACT
ACAAGTGGAAAATAAAGTAAAATCTATAGAACGGAAACTGAGGATAAATGAATGCGCTAGTAATCCTT
GTATGAATGGTGGAACGTGTTACGATCTTTATGAAGGATACGAGTGCCACTGTCCATCAAATTGGGAGG
GGCCAAATTGTATGGCGGATGTAAACGAATGCGTTCGACTGCTAGGAACAGACCTCGGATGTCAAAAT
GGAGCCACTTGTCGTAATCTTCCAGGATCGTATAAATGTGAGTGCGCGGCAGGATATTACGGCCTCCAC
TGCAAGAAAAAGTCTTCAGTTTGCAACACTCAAAATTCCAACGAGCTATGCGGCCACGGCATATGCGT
GAGCAAGCTTGGAACACCGCTCGGTTACACTTGCATTTGCGATCAAGGTTGGCAATCGGAAGGTACTA
ATCCAGCTTGCATCAAGGACGTGGACGAATGCGCTGGAAATCATAGACCGTGCTCCGTGAATCCTTGG
GTTGCTTGCCGAAATGCGCCTGGAACATTCTTCTGCGATTCCTGTCCACGAGGCTACACAGGAAACGG
GTACTATTGCGCCGACATAGACGAATGTCAAGTGAACAACGGAGGCTGCAGCACGTCCCCTCTCGTCC
AATGCATCAATACCATGGGTTCGAGAATGTGCGGCGCCTGTCCTACTGGATACCGTGGCGACGGCGTG
ACATGTGTCTACGTAGGTAGTTGCGCGATCAACAACGGAGGTTGCCATCCATTAGCCACGTGCGTCGA
GAATTCAGCACTTACGAGCGCCTACGTGATCTGTCGATGCCCACCCGGCACAGCGGGCGATGGAATCG
GACCCAACGGTTGTCAATCATCGACCGAGGCGTCTCCCTGCTCCAATAATCCCTGCGTGCACGGCAAG
TGTGCCGCCGTTTCCGGCACCTACTCTTGCACGTGCGATCCTGGATACACCGGGGCAACGTGCAACGT
GAAAATCGACCCTTGCTCCCCGAATCCTTGCAAGAACAACGGCGTCTGCACGATTTCGAACGGCGCG
GCGACCTGCGATTGCCCGTCGACGTACACCGGCACCAGATGCGAAACCCCGCGACAGACATGCGGCG
GTGTTTCACGTAATCCCGTGGGACATCTCGAATTCCCGATAGGCGGGAACGTTTATCAACACGGGCTG
AGCTGTGCCTGGGTGCTGATCACAAACAGCTCCCTCGTGTTGAACGTCACGTTCACCCGTTTCAATCT
GGAGCACTCGACCGACTGCAAGTACGACTTTTTGCAGATACACGATGGTAGGAACGCCGGCAGTCAG
ATGATCGGCAGATTTTGCGGCCAAGAGTTTCCCTACGAGAACAAGAATATAGTGTCCTCTCACAACTCT
CTCTACTTCTGGTTCCACTCCGATAACAGCGTGTCCTACGACGGATTCGCGTTTGAATGGAACAGCGTC
AAGCCCATTTGCGGAGGTAGTTTGACGAACGATTACGGGACGATCAGCTCCCCTGGATCACCCGGAAG
GTATCCGCCGAATAGAGACTGTTATTGGCAGATTACCGTTAAATCCGGGAAGAGAATACAGATCCATTT
CGGCCAATTGATGCTGGAGGAGCATCGAACGTGCGGAGCCGATTTCCTCGAGATCAGCACTATACACG
GGGAACGATTGGGACTTTACTGCAATCACTCGCATCCAGCTCCTCTTATCGTACCCGCCTCCAGCGTCG
TGATCTATTTCCACTCTGACGGTGCTGGCCAAGACTCAGGCTTCCAAATTCACTATTCCGCCATTCAAG
GTCGTCCAGGTTGTAACGAAGTGTACACTTCTCCGTCTGGTATCATAAGAAGCCCTATGTCCGCTGAAC
AGTGGGAAGAACTAGAATGCGAATGGAAGATTCAATTATCCGTTGGAAAACGAATACAAATTTTTTGG
ACCACGTTCGAGCTTTTGAAGAACAATTGTCATATGGAATACGTCGAGATTTATGACGGCGAAACGAG
CGAATCTCCATTGATACAACGATACTGCGGTAGCTCGATTCCACCTTCAATCACATCGAATTCGAACGT
ATTGTTGATAATCTTCAAGTCGTACGCGTATCAAATAGGCGCATTCTCACTTTCGTACAAAACAATATGC
GGCAAGATATTCACCGAAGTGTCAGGCATCATTCAATCGCCTACGTATCCTCTCTTAGAAGTTACGAAG
CAGGTGTGCACCTACGAGATCAGGCAACCGCCGAACAGGAAAATCGTGCTGAAAATATTAGACATAG
ATATCAACAATTCGTTGAGAAGAAAGTGCATTTTCAACTATCTCGATGTGTTCGATGGACCCAACGAGA
ATTCCACCCGCCTCGCCAATCTTTGCAAAACTGCCATTGACTTGACGTATTATTCGACGCACAACGTAA

TGACGCTTAAGTACTCGGGTGCAACGGGGAAACGCGGTTTCATGGCGAATTACACGACGACTGAGATA
GGATGCGGAGGATTGTTCACGGAGCGTAGCGGGACGATTCAATCACCGAGCAACGAAGGCCGTTACA
GAAACAATGAAAACTGCATCTGGACTATACAAGCGCCGATAGGACACGTGATCCATCTTAGCTGGTTAT
CCTTCAATTTGGAGAATAATCGTAATTGTCCACACGACTACGTGAAGATATACGAAAATTTCATGTCGT
CCAATCAGGAGATAATAGGAACATTCTGCGGCACGAAACATCCACCGACCATAATATCACAAGTGAAC
GATATGACGTTAATCTTTCATTCCGACTCGTCGATCATCAACGAGGGATTCATCGCATCGTACATGTTCG
TCGACGCGAGCAAAGTTTGCGGCGGTCATTTCGTTAAACCGATCGGTGTCATCAAGTCGCCAAATTAT
CCCAATCGTTATCCACGCGGGAGGGAATGCGTGTGGGTGATTGAAGCGGCGAACAAGCAAAGGGTGA
TAATCAACGTGGAAAAATTCAACCTGGAGAGGCACACGACTTGCGGTACGGACTATTTGGAAATCAG
AAATGGCGGTTACGAAACCTCTCCGTTAATCGGCAAATTCTGTGGAACCGATATCCCCTCGGAGATCAT
AAGCCAGACAAATCAATTGTACCTGAAATTCGTGAGCCGTTCGAGGATGAGTTATCCCGGATTTAGCAT
AAGATGGGACAGCACGACAGTAGGATGCGGTGGATCTTTGACTGCCGCCCAGGGCGATATCATATCTC
CGAATTATCCGATGCCGTACATGCATCAGGCGGAATGCTATTGGAGGATAGCTGTTGCCGAGGGTAGTT
TGGTACGATTGATAATACTGGACCTTGAATTGGAGCATCACAACAAGTGTAGATACGACTACATCGAGA
TATCGGAGGGGATGAACCGCCGGAACAGCGAAAGATTTTGCGGCAAATCTAGCGCGAAAATCATTCA
GAGAGCGTCCAACATACTGAACATAAAGTTCCATAGTGACTTTACGAATTCCGGCCGCGGTTTCCATCT
CAAATACGAAACACTGTGCCAGAATATCACGATACACAATTATTACGGAATAATAGAATCGCCAAATTT
CCCGTACAAGTACGAGCACAATCTGAACTGCAGCTGGATGATCGACGCCCCCATAGGTAACAAGATCA
ATCTGACGTTCTCCCATTTCGACGTGGAGGGATTGGGCAGGAATAACAGCTGCGAATACGATTATCTGG
ATATATCCGAGGGTATGGATAAAACACCGTCGAAACAACTCGCGAAACTGTGCAACTCAGACGTGCTC
CCTGCAAAAATCCATTCGTCGCAACATCAAGTGTTCTTGAAGTTCATCACCGATTCTTTTATCGCTTTCA
ACGGGTTTCGGCTCGAGTGGATGGTCGATGGTTGCGGTGGACATCTGACCAGACCCTTCGACACGTTC
ACATCACCCGGATATCCGTCTTCGTATCCGACGGACATCGAATGCGAGTGGTTGATCGAGGTCGATTAC
GGGCACAGCATCGAACTGACTCTTCACGATATAAAGATCGAGAAAAAGATAAAGTGTTATTTCGATAA
ATTGCAAATTTACGGGGGCGAGAACGAGCAGGCACCGTTGCTGGTCGAAATCTGTTATTCGTCTAAGC
CTGTAGTGTACACCAGCTTCGGGAACACGATGTTCGTGAAATTCCTGTCCGATGTAACCTACGTGTCGC
GTGGATTCAACGCCAGTTACAAAACCGTTCCTATCACTTGCGGTGGAAGATTTACAAGCGATTCTGGA
ATCATCCACTCGGCCAATTATCCTCAGAATTATCCGAATAAGCAAAATTGCAAGTGGTTGTTCCAAGTC
GATCAAAATTACGTCGTTAATATCACTTTCTTGGACTTCGATATCGAGAACACCGAGAATTGTACCGAC
GATTACGTCAGGATATATGACGGGCCAACGACTGATTCACCAATATTGGGAACGCATTGTCGAAATCAA
TTGCCGCCTTCCTACGTATCGACCAGCAACGAAATGTTACTCGTGATGAGAACGGATTCTTTGATTTCG
GCAAAAGGATTCAAAGCCCAATACCGGAAAGCCTGTGGTGCTCGTATCATTGTGAAGGATCAAGGTTA
CATAGTTCCATACGAAACTTACATCGGCAATTCAGATTACAGCGAGAATTGTACGTGGACATTGATCGC
CGAGAATCTAGACGATCACGTTACCGTGACGTTCACTCATATGGACTATGTCGATGAACTTGATTATTTG
CAATTTTGGTCCAGAGATTGCTTCAACTACATAGAGGTTTTCGATGGTGTGGACACGACTGGGCCATCT
CGAGGCAAATGGTGCAATAAAATCGTACCATTGCCTATAACTAGCAACGGAAACGCTCTCACTGTGCA
TCTGTACAAATTTCACGAGAACTCGATACACTTCTCTCTCGCATATTCCGTGTTAAATTCAGCATGCGGC
GGAAATTACACCGCGTATCACGGAGAAATCGCGTCGCCGAACTACCCGAACAGCTACCCCTTGAACTC
GGAGTGCATCTGGATCCTGGAGAACTCGCCCGGAAACAAGATCAGCCTGTCTTTCAGTCAGTTCAACC
TGCAACAGAGCGAGGATTGCAATCTGGATTACGTGGAGATTCGAGAGGAGAGCGGGATCGGGAAGTT
GATCAGCATTTCCTGCGGGACAAGCGTGGAACCGGTCGAATCTTCCAAACCGCTCTGGATCAAGTTCA
AGAGCGACGGCGATGGCGTGGCGACGGGATTCCGGGCTGAATTCAGCGTGATGGGTGGGAACGAGTT
GAGTGGACCCTCGGGCAAGGTCACCTCTCCCCTTTACCCGATTCCGTACAAAGGAAGAGACACCCTTT
CGTGGAGGATCACCGTCGAATTTCAATGGGCGGTCCGAATCCAGTTTACCGATATATTTATCGAGAATA

GCGACACGTATTGCTTCTCCTACTTCAGGGTGTACGACGGTTACGACAGCAATGCTCCAATTCTGTTGG
AAATATGCGACCTTGACATCCCGGATCCCATCACATCGTCTACCAACGTAATCTACATCGACTTCTCGGT
CGATATCCTCCGGCAGGGTAGTTGGTTCGAATTAAATTGGATTCAGGTGCCGATCGTCAACGATGACAA
CGGCGAGAACATATTCAAGAAACTATCGGAGTGCAACATGATAGTAGAAGTGCAGAACTACACCTACG
ATTTCACTTCGCCCGGCTGGCCCCATGGTTACGACACGTTCCTTCGCTGTAACTGGGTTTTCCTCTCCC
CACCCGGAACTCACTTGAAACTCAGAATTTTAACTATGGATTTGGAGGAAACGGCCGAATGCACGGAC
GACTTCGTCGCTGTTTATTCCGGCAACGCTCTGACGGACGAAAGCAATGCTAATTTGTTGCGGAAATT
GTGCCTGGCGAATTCCACGTTGGTCGATGTTGAGGTGGATAACGTGATGACAGTTAAATTCGAAACGG
ATGGATATAGGAATAACACGGGATTCAGCGCGTTCGTTTCTAAAGAGTGTGGCGGCAAGATGGAAGGT
CCAAACGGAGTGATCGAGGTGAATGAGACTACTACTTGGTCGAAAAGATGGTTGAGACGATTGCAGT
ATAAGGTGAATTGTGAATGGGTCGTCAAAGTGAGACCTGGACGGACAATAGAAGCGAAGATCGTCTC
GATGTCGATACAAAAAGGACCCAATTGGACGTGTACTAATAATTATCTAATGTTGAAGAATGGCGGTGA
CTCTTCCTCGCCTTTCCTTGGCTCCGGTAAATATTGCGGCGACGTAACTCCACCCAGCTTCAAAACTAT
TGGAAATCTTCTGTACGTGAAGACGGAAGGTTTTCGTGACAATATCAACTTCAAACTAACTTACAGAG
AGGTGAGCATGAATTGTGGAGGCGAGTTCCATTTATCGAACAAACAGAAGGAATGGGAGATCAGCAC
GCCGAATTATCCCAACATACCTGCCCCGTATTCCGAGTGCGTATGGACCGTGATGGCGCCAGGCAAGG
AGAGGATATTCATCCATTTCATCGAAAGATTCGATCTGAGCAATACCATTAACTGCGAGAAAGAATACG
TTGAAATAAGAGATGGTGGAACGGAGAAATCCAATCTGCTCGGAAGATTTTGCAAAGATGTGGCGCCT
AGCAGCATGACGAGTACAGGGAATATGATGCGCATCCACTATTTCTCGGACTTGTCCGAGCCGAAAAA
TGGCTTCAAAGCTCTGTTATCCATCAAACACATCTGTGGAGGGATTATCAGAGACGTTAGCGGTGTCAT
CTCGTCGCCCAATTTCCCATTCTTCTATCCAAAGAATCAGACCTGCACTTGGTGGATAACTGCCCCTGC
CCATCACACGTTGAAATTCACGTTCCTGGATATAAATCTACCCGGTCTTCGACGTTGCAAGATCACGGA
TCACGTGCAGATCGACGACGTATTCAAGAATTGGAACGACACCAGCGTGAAGAGGACCGAAATTGGA
ACTTACTGCGGTATGACGATACCCGATCCTATCGAAACAGCGACCAACGAAGCTGTTATAACGTTCCAA
AGCGATAATTTCGAATACGCTGTGTACAAGGGATTCAGTATCAGTTTCAACGCTAGTAACGAAATATGT
GGAGGCGAGCTAACTGCGATGCAGGGCACGATCAAATCGAATGGGTATCCTAATATCGCAACACGTTC
GAGATACTGCGATTGGAGAATCAAATTGCCGAGGGGTTATCACGTGGTCATCGATGTACAAGATTTACC
CGAATATCAAGAAAATTCCCCATTATATTATTTAACGTTCTACAACGATTTCAACTTCAAGTCTAAGATC
AAGATATTAAGACCGAACTCAACCACGAAACGAGTAACCAGTTCTGGCAACACTATGATGATCGGTGC
TTACACGTCATCGGGTTATCGTGGTTTCAAATTGCGTTACTTTGCCGAGGCGCCAGCTCCCTGCGGTGG
AGAGATAAATGAAATGAAGGGTAATCTGTCAGCTCCAAGAGAACTACCCTTCAACGAATCGTCCTATT
TCTGTCAATGGATAATTAAACCACCGGAGAGTTTGTTAAGCAATAACAATACTGGTGTGACATTGTCGA
TAATGGTAACAGGTGTGATTGGAGGGATTCGCGGCCTTGCTTTTACCAAGCTTTGCTTCAATAATCAGT
ACATCTCCTTGAAAGGTATCGGTATGCTTTGCGGAAATTTCACGGAACCAACCTATCTGAGAAGCCCG
GAACTCGTCAACGAATTGAACATAGTGAACGGTACCTATGGAACGTCGATGCGTTTCACTTTGGAATA
CAAATGGCAACGTTGTGGCGGTATATTTCACGGAATGTCGCATGTGATAAAGGCGCCTAAGAACGTATC
GTATCCTATTAATTGCGTCTGGCACGCAAAATATCCGAATAACGAAATAATCAGACTTCATTTCAACCAA
TTACATTTGGGCAGCTGTGATAAGAATTACTTGAGTATCAGGAATGGTGGGCCTTTTTCACCAGAGATC
GGGAAATTCTGCGAAAATTCCCAATCGTACAACTTCACTAGCACGTTTAACGAAATGTGGATCGAGTT
CGTGGCAATCGAGGAGCCGACAGATTTCGAGTTCACCCTGGAACCAGCTAATAACGGATGCGGGGGT
GCTCTGCGTGGTGATAGCCGAGAGATTTCGTCGCCCAACTTCCCCTCGGCGTATCCCAACAACGCGGA
ATGCACTTGGGAAATAACCGCGGACAATGGCTACAGCATCGGCCTAGTATTCGTTGATCGTTACCATTT
AGAAAGCAGCACTAATTGCGAGAAAGATTACGTACAGATATTCAACTGGATCAAGGAAACGGGCGAA
TCCTCCGCTGGAACGTGGAAGGATCTCGGAAAGGTGTGCGGTAGACACACGCCGTTGCCATTTAATTC

GACGAGCAATCGCATGAAAGTAGTTTTCCATTCGAACGAGGCGATCCAAGGCGACGGATTCCGCGCCG
TATGGTTCGAAAATTGCGGTGGAATTTTCGACGTCACCGCTCATCCAAAGGTGATCGTGTCACCTTCGT
ATCCGATTTCGTACCCGCCAAATCTCTTCTGCAATTATACCCTGGTGGCGCCGGGCAAAGACATTTTGG
TCAAATTTATGGATTTCCAGATCGAGCGTAGTCGCAGGGGTTGTCGTTACGACAACGTGACGATTAAAT
ATCAAGATGGATATATGAACGAGGAAAATACCTGGTGCGGGGAAGATAAACCACCCTTGATAAGAGCG
TCAAACGCGGTGGAGATTATATTCAGAACCGATAATTATCTTGCACGAAGTGGTTTTAAATTCAAATATT
TCCTGCATGAATGCGGGGGATTGTTGACAACGCCAGGAGAGATTAAGCCTTTAATGAATGGCAATCAA
TACTTTGGAAGACTAGATTGTACGTGGAAAATTCAAGCACCATCCGACAAGAGCGTCGTCCTTCGTTT
CGAAAGTTTCGACATCGAGTTCAATTTCAATTGTATATTCGATAATCTTCAGATCTATAATGGGTCGGAA
ACATTGGACGAAAACAAAATAGCCGTGTTATGTGGAAACCTTACGGATAACTTGCCTGTCATCAAATC
GAATTCTAATTCCATGGTGCTTAAATTCCACGCGGATGACAGCCGCCATTCCGCTGGATTTTCCTTAAA
AGTACAATTCGTGAAGAGTATCAGCGCGGGTTGCGGGGGCAATATTAATTTAAGTTCGACTTCGAGTC
AATCGTTCAAGACTCAAACAGGTTCGACGTACGATAGCCTGGAGGATTGTCAGTGGATTGTGTCAGCG
GCCGAGGGGAAAAACATCAAGTTCACGATAAATAGCATGGACGTTAGAAACAGTACTAATCGCACCG
GAAACGGGTGCACCGGTGATTACATCGAGATCCGCGATGGAGCCGGACCTTTCAGCGAGCTCCTCGG
AAGATACTGTGGAAACCGACCTCCACCTCCCATATTATCGAGTTCCAACTCACTTTTGATCCGATTTTAC
AGCGATGGAACGGTGGAAGGAGCTGGAGTCAGCGGAACGTTGGAAACAGTAGATGCACTGTGCACTT
ATGTTTCTCCGATAATAAACGGCTCGGGAAACATATTGACTTCACCGAATTATCCGAATAATTACGAGCC
CGGCACAAAATGTCGTTGGACGATAAAGTTTGTGCACTACTTCGAAAGAATGAGAATAAAATTCTTGG
ACTTCGATCTAGCTGATTCTCGCGAATGCAAAGACGATTTCCTTCAGATCACAGATAAAGAGAATCGA
AAATACATCGAGCAAGGACTCGGCCAGGACTTAGTTTGGAGTGGAAATTCGGGCAAATCGTATTATTT
GAATGAATTCATGCCAAAAACCGCGTACAAATATTGTGGCAACAGTCTGCCTCACGATTACTACAGTTA
CAGCACCGAATTCGACTTAACGTTCAACGGTATTAAACCTGGGCACAAAGGTTTCAAGTTGGAGTACA
GCAAGGCGACTTGCGATCGGAATTTCACCGAGTTGCAAGGTCGAATCGTGCACGAGGGTATCGACGA
CTGTTGGATCACGATCACCGTCCCCGAGAATCACACGATCTCTCTATACTTCAATCAATTCATGCTCTAC
GATCAAACCGATTGTACGAAAGCTGGCCTAAATGTGTTCGACGGTGATTTCCATGGTAAACTGATGGC
GTCATTGTGCTCCATAGATACGCCAAGTCCTATTTTCAGTACCGGAAATAAACTGAGTTTACGTTCCTG
GTCCGAGTGGCATTCTAGCTACGAATACTACGATATCACGTACACCACCACGAACGCAGGTCGTGGAT
GTGGCGGTAAAATATTTAATTATGCTGGATCTTTCACATCGCCCATGTATCCCAACGAATACAGGAATAA
TACGATCTGTATTTGGGATGTTAATGTGCCACGTGGACTGAAAGTCGTTCTAACATTCACAGTGTTAGA
CATTGGCTCGAAGAGCACCTGTAACTACGATTACAATATTGTTTCAATCTACGATGTAACGTCTGATGG
AACGGAAGAGTTTGCCACCAGTTATTGCGGAGGGGATGACCCAGCACCGTTCATCGCCACCAGCAAT
CGTTTGATCGTGAAATACATTTCATCCGTGAACAATATCGGAACAGGATGGAGAGCGATCTTTGAGGGT
CGAAATAATTGAGATTTCTATTTCTGAGTAAGATTCTAAGGACAATTTATAAAAATATTTATTTGCGTATG
TATATCTATCGAAAATTAACAGTATATTTATATCAACCGCGCTCCAAGCAAGTGGGTAAAATTAAGACGG
TTTATTGTCGAAAAATTCGTTATTTGTAAATAATGTAACAAGTATTTGATATGCATAGAGATTAAATAAAT
GAAATTTCGTATAA
