# Supplementary material for: Alternative Splicing and Alternative Polyadenylation-Regulated Cold Stress Response of Apis cerana
Source: Insects. 2024 Dec 19;15(12):1006. doi: 10.3390/insects15121006 (PMC11677483; doi:10.3390/insects15121006)
Supplement: Supplementary file 1 [file insects-15-01006-s001.zip › insects-3356587-supplementary tables.pdf]

insects

Supplementary Materials

Alternative Splicing and Alternative Polyadenylation-Regulated Cold Stress Response of *Apis cerana*

Yuanchan Fan [1,†], Dan Yao [2,†], Jinmeng Ma [1], Fangdong You [3], Xiaoping Wei [2,*] and Ting Ji [1,*]

Table S1. Summary of Pacbio sequencing data.

day-old	Treatment group	Raw reads	Average read lengths (bp)	N50 (bp)
*Ac*3d	CK	537 698	132 366	210 799
*Ac*3d	T	387 552	137 915	218 149
*Ac*3d	TR	352 722	128 308	197 033
*Ac*10d	CK	329 469	129 800	197 011
*Ac*10d	T	310 214	130 010	197 873
*Ac*10d	TR	724 422	98 397	169 861
*Ac*21d	CK	418 947	140 398	217 070
*Ac*21d	T	324 980	130 010	203 470
*Ac*21d	TR	959 759	141 959	218 264

Table S2. Primers for PCR & 3'RACE.

Primer name	Primer sequence (5'-3')	Purpose
LOC107996313_novel06-F	CCTTTGAAAGTTTTACCG	PCR
LOC107996313_novel0-R	AATAGTGTTGTTCGGGTT	
LOC107996313_novel10-F	TAATAGAATGAGGGACGG	PCR
LOC107996313_novel10-R	TGGATGAAAAAGTAACGA	
LOC108000663_novel06-F	CAAAGGGTCCACAAAATG	PCR
LOC108000663_novel06-R	GGATGACGAGCTGCTAAA	
LOC107995758-F	ATAGCATAAAAAAATAAAAC	3'RACE
LOC107995758-R	TTTATTGGTATTGTCATTAT	
LOC107998211-F	GGCTTACAGATGGATTATTG	3'RACE
LOC107998211-R	ATTTTTCGTTTATTATGATT	
LOC108003581-F	GTCGTTCTAACATTCACAGT	3'RACE
LOC108003581-R	GAGTAAGATTCTAAGGACAA	

Table S3. Summary of AS type.

day-old	Treatment group	Raw reads	Average read lengths (bp)	N50 (bp)
*Ac*3d	CK	537 698	132 366	210 799
*Ac*3d	T	387 552	137 915	218 149
*Ac*3d	TR	352 722	128 308	197 033
*Ac*10d	CK	329 469	129 800	197 011
*Ac*10d	T	310 214	130 010	197 873
*Ac*10d	TR	724 422	98 397	169 861
*Ac*21d	CK	418 947	140 398	217 070
*Ac*21d	T	324 980	130 010	203 470
*Ac*21d	TR	959 759	141 959	218 264
